# The Association of Hypertensive Disorders in Pregnancy with Offspring Kidney Function from Adolescence into Young Adulthood

**DOI:** 10.21203/rs.3.rs-8032514/v1

**Published:** 2025-11-19

**Authors:** Jo A. Kelly, Mark C. Chapell, Elizabeth T. Jensen, Keia R. Sanderson, Christopher L. Schaich, Hossam A. Shaltout, Andrew M. South

**Affiliations:** Wake Forest University School of Medicine; Wake Forest School of Medicine: Wake Forest University School of Medicine; Wake Forest School of Medicine: Wake Forest University School of Medicine; UNC-Chapel Hill: The University of North Carolina at Chapel Hill; Wake Forest School of Medicine: Wake Forest University School of Medicine; Wake Forest School of Medicine: Wake Forest University School of Medicine; Wake Forest School of Medicine: Wake Forest University School of Medicine

**Keywords:** Pediatric nephrology, pediatrics, organogenesis, pregnancy complications

## Abstract

**Objective:**

To estimate the association of hypertensive disorders in pregnancy (HDP) with offspring kidney function in adolescence and young adulthood in individuals born preterm with very low birth weight.

**Study Design:**

Secondary analysis of data from a prospective cohort study of individuals born preterm with very low or extremely low birth weight (< 1500 g). The 213 participants were assessed at 14–15 and 19–23 years of age. Outcomes were estimated glomerular filtration rate (eGFR) and first-morning albumin-to-creatinine ratio (ACR). We estimated the relationships with multivariable generalized linear models informed by directed acyclic graphs. Causal mediation analysis was also performed to evaluate if adolescent kidney function indirectly mediated the effect of maternal HTN on young adulthood kidney function.

**Results:**

37% (*n* = 79) were exposed to HDP. Kidney function in adolescence was associated with kidney function in young adulthood (eGFR adjusted *β* 0.48 mL/min/1.73 m^2^, 95% CL 0.21–0.75; ACR adjusted *β* 0.19 mg/g, 95% CL 0.15–0.24). There was no significant association between HDP and kidney function in both adolescence and young adulthood. There was also no statistically significant causal mediation effect of HDP on young adulthood kidney function indirectly through adolescent kidney function.

**Conclusion:**

We found no significant association between HDP and offspring kidney function in adolescence and young adulthood, and no evidence of mediation through adolescent kidney function. Next steps in this research may look to investigate the relationship of severity of prematurity on offspring kidney function, as HDP are a factor in premature births and the degree of prematurity.

## INTRODUCTION

Hypertensive disorders of pregnancy (HDP) are associated with significant health complications for both the mother and offspring [1]. These disorders include chronic hypertension, gestational hypertension, and preeclampsia-eclampsia [1]. Several studies have shown that youth exposed to preeclampsia in utero are more likely to be hospitalized during their lifetime up to 27 years old, along with having a higher blood pressure and body mass index in childhood through early adulthood [2]. However, the associations with chronic or gestational hypertension are not clear. In addition, preeclampsia increases the risk of preterm birth [1]. Preterm birth is associated with a higher risk of adverse health outcomes and an increased risk of mortality in early childhood through young adulthood [3]. Furthermore, youth who are born preterm have a higher risk of hypertension and possibly kidney disease later in childhood and adolescence [4].

While there have been several extensive investigations into premature offspring’s health outcomes, especially cardiovascular outcomes, little research has focused on the change in kidney function over time into adulthood. The goal of this analysis is to better understand the association of HDP with future offspring kidney function in adolescence and young adulthood in a well-characterized long-term birth cohort of individuals born preterm with very low or extremely low birth weight. Our aim is to estimate the direct effect of HDP on offspring kidney function in young adulthood and the indirect effect mediated through adolescent kidney function.

## METHODS

### Study Design

This is a secondary longitudinal analysis of data collected in a long-term prospective cohort study of individuals born with very low or extremely low birth weight (birth weight < 1500 g), the Prenatal Events-Postnatal Consequences (PEPC) study. Participants were initially recruited at age 14 years (PEPC1) and were assessed over a series of three study visits. Participants were then recruited into the second iteration of the study starting at age 18 years (PEPC2) and were assessed over a series of two study visits. Due to sample size attrition between PEPC1 and PEPC2, 28 individuals who were not enrolled in the PEPC1 study but met inclusion criteria and lacked exclusion criteria were also recruited and enrolled in the PEPC2 study. The institutional review board approved the study, and written informed consent and assent were obtained from parents or legal guardians and participants at PEPC1 and PEPC2. Further details on the PEPC study design have been previously published [4].

### Study Population

**Figure 1:**
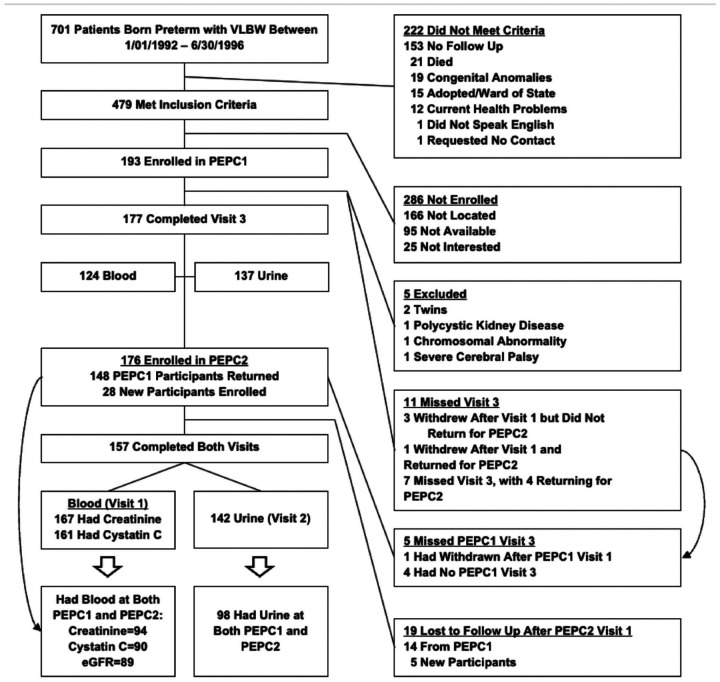
Exclusion Criteria Flowchart for PEPC1 and PEPC2. 213 participants used in this study are composed of the 193 enrolled in PEPC1 (except the 5 excluded and 3 that withdrew), and the 28 new participants in PEPC2.

Inclusion criteria were singleton births at Forsyth Medical Center in Winston Salem, North Carolina, from January 1, 1992, to June 30, 1996, birth weight < 1500 g, follow-up data through 1-year corrected age, successful contact at 14 years old for PEPC1, and successful contact at 18–23 years old for PEPC2. Exclusion criteria were significant congenital malformations, primary health conditions in adolescence or young adulthood, and inability to complete study procedures. For the primary analysis, we excluded participants lacking blood and urine samples for kidney function analysis at PEPC1 and PEPC2 (Fig. 1).

### Study Data and Procedures

Study staff retrospectively recorded antenatal and infant data from health records and research databases. The study staff administered questionnaires to parents/legal guardians at the first study visit in PEPC1 and again in PEPC2 for new participants. The original study design collected race only as self-reported Black or non-Black. In this analysis, we conceptualized race as a social construct, not as a proxy for biological (e.g., inherited) traits, ancestry, socioeconomic status (e.g., social risk factors), or racism.

For descriptive purposes, we classified participants by gestational age at birth per guidelines: (i) moderately preterm for gestational age ≥ 32 weeks to < 34 weeks; (ii) very preterm for ≥ 28 weeks to < 32 weeks; and (iii) extremely preterm for < 28 weeks [5]. For descriptive purposes, we classified participants’ birth weight per guidelines: (i) very low birth weight for birth weight ≥ 1000 g to < 1500 g and (ii) extremely low birth weight for birth weight < 1000 g [6].

In PEPC1, participants’ blood samples and first-morning urine samples (collected at home) were obtained at the third study visit; these collections were optional in the study design. Serum creatinine was measured to estimate the glomerular filtration rate (eGFR). eGFR was calculated using the original Schwartz equation k × height (cm) / serum creatinine (mg/dL), with k = 0.55 for females and k = 0.7 for males [4]. We chose this equation because it is validated in a general pediatric population lacking known chronic kidney disease and thus is less likely to underestimate kidney function [7]. Urinary albumin and creatinine were measured from the first-morning urine sample. Those values were then used to calculate the albumin-to-creatinine ratio (ACR), and albuminuria was defined as a ratio > 30 mg/g [8]. The creatinine was analyzed using a modified Jaffe assay traceable to isotope mass spectrometry, which was later updated during the study period and a correction value of 1.06 was applied to the relevant original values [4]. In PEPC2, participants’ blood samples were obtained at visit 1, while the first-morning urine sample was collected at home and brought by the participant to visit 2. Serum and urine creatinine were measured with a modified Jaffe assay traceable to isotope mass spectrometry, and serum cystatin C was measured with an ELISA [8]. eGFR was estimated using the 2021 CKD-EPI equation for creatinine and cystatin C. The ACR was calculated, and albuminuria was defined the same as mentioned above in PEPC1. Our exposure was HDP which included chronic hypertension, gestational hypertension, or preeclampsia; our original study design’s data collection methods did not reliably distinguish between these conditions [9]. These conditions were reported via questionnaires. Our outcomes were eGFR, ACR, and albuminuria at PEPC2. Our mediators were eGFR, ACR, and albuminuria at PEPC1.

### Statistical Analysis

To summarize the data, we reported frequencies and proportions and utilized measures of central tendency, including mean with *SD* and median with interquartile range. We also applied the chi-squared test, Fisher exact test, t-test, and Wilcoxon rank-sum test for between-group comparisons in Table 1, as indicated by whether test assumptions were met. A two-sided alpha level of less than 0.05 was indicative of statistical significance. SAS Enterprise Guide software version 7.1 for Windows (SAS Institute Inc., Cary, NC) and R 4.2.3 (2023) were used for all data analysis.

We first used bivariate and multivariable generalized linear models to estimate the associations of our exposures with our outcomes. No participants at PEPC1 or PEPC2 had an eGFR below 90 mL/min/1.73 m^2^, and the proportion with albuminuria was very low at both PEPC1 and PEPC2 (7% and 5%). Thus, we did not analyze these outcomes as dichotomous variables in our regression models due to rare-event bias and anticipated imprecision in our effect size estimates. As our remaining primary outcomes were all measured on a continuous scale, we used an identity link function and normal distribution to estimate *β* with 95% CL. We first estimated the associations of HDP with kidney function assessed in adolescence at PEPC1 with eGFR and then with urine ACR in separate models. Next, we estimated the associations of HDP with kidney function assessed in young adulthood at PEPC2 with eGFR and then with urine ACR in separate models. Secondarily, we estimated the associations of kidney function in adolescence at PEPC1 with kidney function in young adulthood at PEPC2 for both eGFR and urine ACR in separate models to confirm the expected within-participant correlation of kidney function over time. We initially performed complete case analyses.

**Figure 2:**
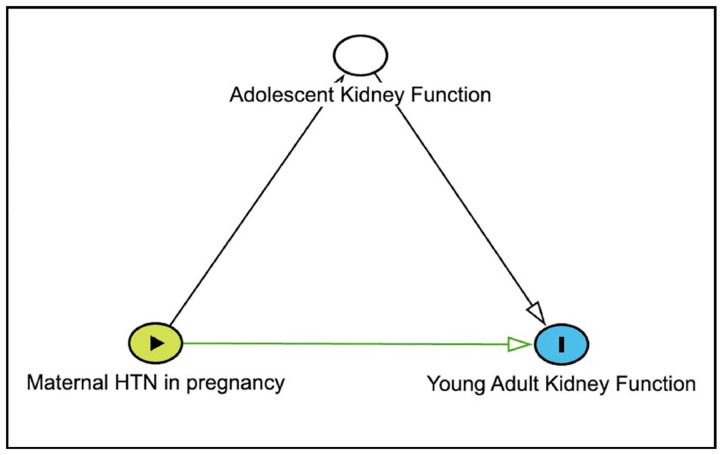
Directed Acyclic Graph (DAG) Illustrating the Causal Effect of Maternal HTN in Pregnancy on Young Adult Kidney Function. The green node is the exposure, Maternal HTN in pregnancy. The blue node is the outcome, Young Adult Kidney Function, and the white node is the mediator, adolescent kidney function.

Applying a causal inference framework to our analytic plan, we developed graphical causal models of the relationships among our exposure, mediators, and outcomes using directed acyclic graphs (DAGs) a priori to inform the minimally sufficient adjustment sets included in each multivariable model to close biasing, non-causal paths (Fig. 2) [10, 11]. The DAGs were informed by the literature and our clinical and epidemiological expertise. Our adjustment sets included maternal smoking during pregnancy for the eGFR and urine ACR models at PEPC1 and PEPC2. For the model estimating the association of adolescent kidney function at PEPC1 with young adult kidney function at PEPC2, the adjustment sets included acute kidney injury in the Neonatal Intensive Care Unit, height at PEPC2 visit 1, and obesity at PEPC2 visit 1. The association between adolescent and young adult urine ACR was additionally adjusted for sex.

We performed a causal mediation analysis to estimate the direct effects of HDP (our exposure) on kidney function at PEPC2 (our outcome) and the indirect effects mediated by kidney function at PEPC1 (our mediator) [12]. We performed separate models for eGFR at PEPC1 and PEPC2 and then ACR at PEPC1 and PEPC2. We applied the following assumptions to our models, guided by our DAGs: (1) the effects of the exposure on each outcome were unconfounded conditional on our adjustment set; (2) the effect of the mediator on each outcome was unconfounded conditional on the adjustment set and on the exposure; (3) the effects of the exposure on each mediator were unconfounded conditional on the adjustment set; and (4) none of the mediator-outcome confounding factors were themselves affected by the exposure. We assessed for an interaction between the exposure and each mediator by adding an interaction term to the models and applied VanderWeele’s 4-way effect decomposition method [13].

## RESULTS

### Study Population Characteristics

Of the sample in this cohort, 193 were initially enrolled in PEPC1. Five were excluded for anomalies including a set of twins, polycystic kidney disease, chromosome abnormality, and severe cerebral palsy. Additionally, three of the original PEPC1 group withdrew after their first visit. An additional 28 participants were added to the cohort for PEPC2. This made our sample size for this analysis a total of 213 participants (Fig. 1).

Of the 213 included participants, 113 (53%) were female, 89 (42%) were Black, and 79 (37%) were exposed to HDP (Table 1). The mean eGFR was 130.2 mL/min/1.73 m^2^ at PEPC1 (*n* = 124) and 168.7 mL/min/1.73 m^2^ at PEPC2 (*n* = 159). The median ACR was 5.3 mg/g at PEPC1 and 3.1 mg/g at PEPC2 (Table 2). Kidney function in adolescence was associated with kidney function in young adulthood (eGFR adjusted *β* 0.48 mL/min/1.73 m^2^, 95% CL 0.21 to 0.75; ACR adjusted *β* 0.19 mg/g, 95% CL 0.15 to 0.24) (Table 2).

### Association of HDP and Kidney Function at Adolescence and Young Adulthood

There were no statistically significant associations between HDP and kidney function values in adolescence or young adulthood (Table 2). At PEPC1, the eGFR was 3.4-mL/min/1.73 m^2^ lower in the exposed group (adjusted 95% CL −14.2 to 7.3). The urine ACR at PEPC1 was 5.6-mg/g lower in the exposed group (adjusted 95% CL −17.3 to 6.2). At PEPC2, the eGFR was 0.4-mL/min/1.73 m^2^ higher in the exposed group (adjusted 95% CL −7.9 to 8.7). The urine ACR at PEPC2 was 0.5-mg/g lower in the exposed group (adjusted 95% CL −7.6 to 6.6).

### Causal Mediation Analysis

There was no statistically significant causal mediation effect from adolescent kidney function on young adulthood kidney function (Table 2). The average causal mediation effect by adolescent kidney function for eGFR was − 0.2 mL/min/1.73 m^2^ (95% CL −3.0 to 2.7) and for urine ACR was − 1.4 mg/g (95% CL −4.9 to 1.8).

## DISCUSSION

In this analysis of long-term kidney function in young adults born preterm with very low or extremely low birth weight, exposure to maternal HDP was not associated with offspring kidney function. Furthermore, kidney function in young adulthood was not mediated by earlier kidney function in adolescence. These findings are similar to that observed in Keijzer-Veen et al.’s study done in 2007 that examined young adult kidney function at 19 years old in those exposed to intrauterine growth restriction who were born at < 32 weeks [14]. They also did not find a difference in kidney function between exposure groups [14]. This study is relevant to our study as HDP have been shown to be associated with growth restriction [15] and can negatively impact nephrogenesis [16]. There may be a greater risk of alterations to nephrogenesis during the third trimester, as preeclampsia has the highest incidence in the third trimester [16].

Previous research can provide possible explanations for the lack of association between HDP and offspring kidney function. For one, multiple other factors besides HDP can impact offspring kidney function, such as maternal body weight and maternal and offspring nutrition [17]. Additionally, Wu et al. in 2024 demonstrated that kidneys in premature offspring can go through a period of catch-up growth where the nephrons are able to develop and allow the offspring’s kidneys to function normally by 3 years of age [18]. Previous studies that examined the effect of HDP on offspring outcomes found that there is a higher rate of adverse fetal outcomes when there are multiple HDP superimposed onto each other as opposed to one individually [19]. Bánhidy et al in 2012 showed that there was a higher risk of renal dysgenesis in offspring with mothers who had preeclampsia superimposed on chronic HTN as opposed to women who had the sole diagnosis of preeclampsia [20]. Yang et al. in 2022 demonstrated that there was a higher risk of adverse fetal outcomes in women with elevated blood pressure and superimposed preeclampsia, as opposed to women with only elevated blood pressure [19]. These studies reflect that there are multiple factors that can impact offspring outcomes and long-term kidney function, whereas our work specifically focused on the impact of HDP on kidney function as opposed to integrating other possible factors.

A strong point for this study is the well-characterized prospective longitudinal cohort with long-term follow up that specifically recorded kidney function values at follow-up visits over several years. As previously mentioned, while other studies have examined the long-term impact of maternal HTN on the function of other organ systems, few have specifically evaluated its association with kidney function long term. However, a main limitation of this study is that there are no specific criteria that allowed us to differentiate between the various etiologies of maternal HDP. This would be helpful since preeclampsia may be the most relevant exposure, especially if superimposed with chronic HTN [21].

Future research needs to continue to investigate if differences in kidney function manifest in older adulthood as this cohort and others age, as differences in kidney function and risk of chronic kidney disease may manifest in mid to older adulthood. In addition, differentiating between the different maternal HDP will be important to evaluate their individual impact on the offspring’s kidney function. As those disorders have different pathophysiologic mechanisms for how they affect the fetus, it is essential to evaluate their effects separately [19].

Furthermore, it has been demonstrated that neonates born at earlier stages of preterm gestation are at higher risk for more health complications later in life [22]. It would, therefore, be justified to examine if the weeks of gestation at delivery had an association with adolescent and young adult kidney function.

## CONCLUSION

We did not observe differences in kidney function during adolescence or early adulthood in individuals born preterm with very low or extremely low birth weight who were exposed to HDP. Additionally, we did not find that adolescent kidney function mediated the association between HDP and young adult kidney function. Future research should continue to investigate the change in kidney function into middle and older adulthood among affected individuals who may be at higher risk of chronic kidney disease.

## Supplementary Material

Supplementary Files

This is a list of supplementary files associated with this preprint. Click to download.

- PEPCKellymHTNTABLE1.pdf
- PEPCKellymHTNTABLE2.pdf

Table 1 and 2 are available in the Supplementary Files section.
